# A diverse satellite DNA repertoire in *Limnoperna fortunei*: insights into genome structure and chromosomal organization

**DOI:** 10.3389/fmolb.2025.1733652

**Published:** 2026-01-07

**Authors:** Jonathan Pena Castro, Tiago Marafiga Degrandi, Allan Oliveira Leal, Augusto Luiz Ferreira Júnior, Mara Cristina de Almeida, Roberto Ferreira Artoni

**Affiliations:** 1 Laboratório de Genética e Evolução, Departamento de Biologia Estrutural, Molecular e Genética, Programa de Pós-Graduação em Biologia Evolutiva, Universidade Estadual de Ponta Grossa, Ponta Grossa, Brazil; 2 Programa de Pós-Graduação em Genética Evolutiva e Biologia Molecular, Universidade Federal de São Carlos, São Carlos, Brazil

**Keywords:** chromosome mapping by FISH, cytogenomics, genome organization, invasive species, meiosis, repeatome

## Abstract

**Background:**

The Golden Mussel, *Limnoperna fortunei*, represents one of the most successful aquatic invaders in South America, causing significant ecological and economic impacts. Repetitive DNA sequences, particularly satellite DNAs (satDNAs), play crucial roles in genome architecture and evolution, yet the satDNA landscape of this invasive species remains largely uncharacterized. This study aimed to comprehensively analyze the satellitome of *L. fortunei* using integrated computational and cytogenetic approaches.

**Methods:**

We employed a read-clustering approach (RepeatExplorer2) to identify satDNA families from short-read genomic data. The identified families were then mapped onto the chromosome-level reference genome *in silico* to determine their chromosomal distribution. The physical localization of two representative satDNA families with contrasting distribution patterns was validated through fluorescence *in situ* hybridization (FISH) on meiotic chromosomes.

**Results:**

Our analysis identified 129 distinct satDNA families, which were estimated to comprise approximately 9.1% of the genome based on read clustering. Subsequent *in silico* mapping successfully localized 126 of these families to the reference genome, where they collectively represented approximately 5.3% of the assembled chromosomes. Most families showed low divergence levels (0%–5% Kimura substitutions), suggesting recent amplification events. While most satDNAs were distributed across all 15 chromosomes, FISH analysis of two distinct families revealed contrasting distribution patterns: LfoSat025 showed preferential localization to pericentromeric regions, while LfoSat004 exhibited localized enrichment in specific chromosomal regions, demonstrating diverse organizational strategies within the satellitome. Meiotic analysis revealed normal chromosome pairing (15 bivalents), with no evidence of differentiated sex chromosomes, consistent with the species’ predominantly gonochoristic reproductive mode.

**Conclusion:**

This study provides the first comprehensive characterization of the *L. fortunei* satellitome, revealing a diverse and recently amplified repetitive landscape. The discrepancy between the genome-wide abundance (9.1%) and the mapped abundance (5.3%) highlights the challenges of assembling repetitive regions and underscores the value of using complementary methodologies. The identification of chromosome-specific satDNA markers establishes a foundation for developing molecular tools for invasion monitoring, including population genetic analyses to trace invasion routes and identify source populations. These findings contribute to understanding the role of repetitive DNA in genome evolution and the adaptive success of invasive species.

## 1 Introduction

The golden mussel, *Limnoperna fortunei* (Dunker, 1857), is a bivalve mollusk native to Southeast Asia that has become one of the most aggressive aquatic invaders in South America since its accidental introduction through ship ballast water in the 1990s (Pastorino et al., 1993; Darrigran and Pastorino, 1995). Following its establishment in the Rio de la Plata estuary, this species has rapidly colonized diverse freshwater systems across Brazil, Argentina, Uruguay, and Paraguay, causing extensive ecological disturbances and economic losses amounting to millions of dollars annually (Darrigran and Damborenea, 2011; Boltovskoy, 2015). The remarkable invasive success of *L. fortunei* has been associated with its broad phenotypic plasticity, efficient dispersal capacity, and rapid adaptation to novel environments.

Repetitive DNA sequences, once regarded as “junk DNA,” are now recognized as key components of genome architecture, influencing gene regulation, chromosomal organization, and evolutionary dynamics (Biscotti et al., 2015; Bourque et al., 2018). Among these elements, satDNAs represent tandemly repeated sequences that can occupy substantial portions of eukaryotic genomes and play essential roles in heterochromatin formation, centromere function, and meiotic chromosome pairing (Shatskikh et al., 2020). These sequences are also frequently involved in processes of rapid genomic change and may facilitate adaptation under environmental stress—a condition often faced by invasive species (Stapley et al., 2015; Schrader and Schmitz, 2019).

Genomic analyses of *L. fortunei* have revealed that approximately 32% of its nuclear genome consists of repetitive DNA, including about 9% of LINE-type transposable elements (Uliano-Silva et al., 2018). However, a substantial portion of this repetitive fraction remains unclassified, which may result from various factors including species-specific variants, genome assembly gaps, complex repeat structures, and methodological limitations in repeat detection algorithms, particularly challenging in bivalve genomes (Chen et al., 2025). Studies focused satDNAs in bivalves, encompassing 48 species from the Mytilidae, Ostreidae, and Veneridae have identified a total of 26 distinct satDNA families (Šatović et al., 2018). Among these groups, oysters (Ostreidae) exhibit the highest satDNA abundance, with counts ranging from 33 families in *Crassostrea virginica* to 61 in *C. angulata*, representing a genomic contribution of approximately 6%–7%. In *C. gigas*, satellitome analysis revealed an unusual, highly scattered organization of relatively short satDNA arrays across the whole genome (Tunjić-Cvitanić et al., 2021; Tunjić-Cvitanić et al., 2024).

From a cytogenetic perspective, *L. fortunei* presents a diploid number of 2n = 30 chromosomes, with a karyotype composed of 10 pairs of metacentric chromosomes, four submetacentric chromosomes, and one subtelocentric pair, resulting in a fundamental number (NF) of 60 (Ieyama, 1996). Notably, there is no evidence of morphologically differentiated sex chromosomes (Reis et al., 2023). Although the gonadal morphology of this species has been described (Dei Tos et al., 2016), its meiotic behavior of chromosomes during gametogenesis remains unknown. In this context, the present study investigates the composition and chromosomal distribution of satDNAs in the *L. fortunei* genome. By integrating genomic and cytogenetic analyses, we aim to elucidate the structural and functional roles of satDNAs within the satellitome in chromosomal stability and species adaptation.

## 2 Materials and methods

### 2.1 Sample collection and maintenance

Specimens of the *L. fortunei* were collected from the Iguaçu River basin, at the locality of Capanema, Paraná State, Brazil (25° 30′ 29.7″S, 53° 40′ 07.7″W). After collection, the organisms were transported under controlled conditions to the laboratory at Universidade Estadual de Ponta Grossa, Paraná, located approximately 473 km from the sampling site. In the laboratory, mussels were maintained in aerated aquaria with controlled temperature (26 °C) to ensure their survival and acclimatization. Individuals were fed daily with a mixture of microalgae, ensuring adequate nutritional support until the experimental procedures were performed. All procedures were conducted in accordance with institutional guidelines for invertebrate research.

### 2.2 Mitotic chromosomes

Mitotic chromosomes were obtained from branchial and gonadal tissues of 30 individuals (15 males and 15 females) following a modified protocol of (Ebied and Aly, 2004). Mitotic arrest was induced using 0.005% colchicine solution for 72 h at 26 °C. Samples were subjected to hypotonic treatment with 0.075 M KCl for 90 min at 28 °C and subsequently fixed three times in methanol:acetic acid (3:1). The material was dissociated in 50% acetic acid and the resulting cell suspension was dropped onto heated slides (35 °C–40 °C). Finally, slides were stained with 10% Giemsa solution (pH 6.8) for 6 min. Analyses were conducted using light microscopy (100×) for karyotyping.

### 2.3 Meiotic chromosomes

Meiotic chromosome preparations were obtained from gonadal tissues of 30 adult males and females (15 males and 15 females) following a modified protocol of (Almeida et al., 2000). Dissected gonads were subjected to hypotonic treatment in deionized water for 1 h at room temperature and subsequently fixed in Carnoy I (methanol:acetic acid, 3:1) for 30 min. Fixed material was stored in fresh Carnoy I at 4 °C until use. For slide preparation, gonadal fragments (∼3 mm) were macerated in 45% acetic acid, spread onto slides, and dried on a heated plate (35 °C–40 °C). The chromosomal preparations were stained with 3% Giemsa solution (47 mL of distilled water, 1.5 mL of Giemsa, and 1.5 mL of phosphate buffer pH 6.8) for 12 min. Meiotic cells were analyzed and photographed using light microscopy (100×) and epifluorescence microscopy for FISH using an Olympus BX41 epifluorescence microscope equipped with a DP-71 CCD camera.

### 2.4 Nuclear DNA content analysis

Nuclear DNA content analysis was performed using a CyFlow® Ploidy Analyser flow cytometer (Sysmex, Germany) to compare genome size between males and females of *L. fortunei*. For each individual, branchial and gonadal tissues were dissected and mechanically fragmented in a lysis buffer containing Triton X-100. The resulting suspensions were filtered through a 30 µm nylon mesh to remove debris and ensure homogeneity of nuclei. The samples were stained with 4′,6-diamidino-2-phenylindole (DAPI, 4 μg/mL) and kept on ice until analysis. As an internal reference, fibroblasts and sperm cells from the fish *Astyanax mexicanus* were included in each run, a species with a genome size previously established by flow cytometry and sequencing approaches, ensuring reliable calibration of fluorescence intensities. For each sample, at least 5,000 nuclei were measured, with three independent replicates per individual. Fluorescence histograms were processed using FlowMax® software (Sysmex).

### 2.5 Genomic DNA extraction and sequencing

Total genomic DNA was extracted from an adult female collected from the Iguaçu River basin using the DNeasy Blood & Tissue Kit (Qiagen, Germany) following manufacturer’s recommendations. DNA quality and concentration were verified by spectrophotometry (NanoDrop 2000; Thermo Fisher Scientific) and 1% agarose gel electrophoresis, ensuring A260/280 ratios between 1.8 and 2.0. DNA libraries were constructed using the TruSeq DNA PCR-Free Library Preparation Kit (Illumina, USA) to minimize amplification bias. Libraries were sequenced in paired-end mode (2 × 150 bp) on the Illumina HiSeq 2000 platform, generating approximately 2.3 Gb of raw data with ∼1.4× genome coverage.

### 2.6 Satellitome analysis

#### 2.6.1 Read processing and quality control

Raw reads were initially assessed using FastQC v0.11.9 and filtered for minimum quality scores of Q > 30. Low-quality bases (trim <20) and residual adapters were removed using Trimmomatic v0.39 with parameters “SLIDINGWINDOW:4:20″ and “MINLEN:50”. High-quality reads were then subsampled to create libraries of 1 million paired-end reads (∼1M paired-end reads) using Seqtk v1.3 (seqtk sample -s100 input.fastq 1000000). Each sublibrary corresponded to approximately 0.2× estimated genome coverage, meeting RepeatExplorer2 pipeline requirements for repeat detection in intermediate-sized genomes.

#### 2.6.2 RepeatExplorer2 analysis

Sublibraries were uploaded to the Galaxy platform (https://repeatexplorer-elixir.cerit-sc.cz/galaxy/) and submitted to the RepeatExplorer2 pipeline (Novák et al., 2013). RepeatExplorer2 performs graph-based clustering analysis of reads, identifying characteristic clusters of repetitive families, including satDNAs families. Each candidate satellite cluster was extracted as a preliminary consensus sequence (satDNA catalog). To increase detection rates of low-abundance families, multiple iterative rounds were conducted using RepeatExplorer2, as described by (Ruiz-Ruano et al., 2016), reapplying the protocol to unclassified reads from previous rounds.

### 2.7 *In silico* satDNA localization

The *in silico* analysis was conducted to investigate the chromosomal distribution of the identified satDNA families. Consensus sequences from the satDNAs families were used as queries in BLASTn (e-value ≤ 1e-05, minimum identity 80%, minimum overlap 80% of query length, with overlapping hits merged to prevent double counting) searches against the chromosome-level genome assembly of *L. fortunei* (assembly xbLimFort5), available at the National Center for Biotechnology Information (NCBI). For each detected satDNA copy, the corresponding chromosome and genomic coordinates were annotated. These data were then employed to construct ideograms representing both chromosome length and the physical positions of the satDNA families. Ideograms and positional visualizations were generated using custom Python scripts developed with artificial intelligence–assisted tools (Manus ver. 1.5). Subsequently, we quantified the copy number of each satDNA family per chromosome based on discrete BLASTn hits. It is important to note that this copy number metric is distinct from the genome-wide abundance percentage estimated by RepeatExplorer2, which is based on read clustering. This allowed us to identify families with potential chromosome-specific distributions and compare abundance patterns across methods.

For FISH validation, two satDNA families were selected based on chromosomal distribution patterns predicted by *in silico* mapping, representing contrasting organizational strategies. LfoSat025 was selected for its pattern of localized enrichment on specific chromosomes, suggesting chromosome-specific pericentromeric localization. LfoSat004 was selected to represent pan-chromosomal satDNAs, as it showed localized enrichment across all 15 chromosomes, demonstrating that broad chromosomal distribution can be associated with regional specificity. The selection strategy prioritized families showing localized enrichment over those with highly dispersed distribution, as the former are more amenable to cytogenetic visualization and typically yield clearer FISH signals. Additionally, monomer size was considered as a technical criterion, with both selected families having intermediate-length monomers (668 bp for LfoSat025, 1,783 bp for LfoSat004) that facilitate efficient PCR amplification and probe labeling.

### 2.8 Fluorescence *in situ* hybridization (FISH) and probe preparation

FISH experiments were conducted to determine the chromosomal localization of telomeric sequences and to validate our computational predictions for a selected high-copy-number satDNA family, LfoSat004, and LfoSat025, which showed a pattern of local enrichment. The probes of Telomeric and SatDNA sequences were amplified by polymerase chain reaction (PCR) using primers Telomeric (F: 5′- TTAGGGTTAGGGTTAGGGTTAGGGTTAGGG-3′, R: 5′- CCCTAACCCTAACCCTAACCCTAACCCTAA - 3′), TM = 55 °C, LfoSat25: (F: 5′- ACTGACGCCAAACTAAGCCA-3′, R: 5′- TGCGTTTGAAGGTGCAATGT-3′) TM = 56 °C and LfoSat004 (F: 5′- TGTCGATGGAAGTGGTAAGCC-3′, R: 5′- AAGTGTTCGGTGTGACAGGG-3′) TM = 56 °C, designed from previously identified consensus sequences of the target satDNAs families. Each PCR reactions were carried out in a total volume of 50 µL containing genomic DNA, 0.2 mM of each dNTP, 1× PCR buffer, 1.5 mM MgCl_2_, 0.5 µM of each primer, and 1 U of Taq DNA polymerase. The cycling conditions consisted of an initial denaturation at 94 °C for 3 min, followed by 30 cycles of 94 °C for 30 s, annealing at 55 °C–56 °C for 30 s (depending on primer Tm), and extension at 72 °C for 1 min, with a final extension at 72 °C for 5 min. PCR products were labeled with 0,02 mM digoxigenin-dUTP (Roche) nucleotides via PCR incorporation.

FISH experiments were conducted on both mitotic and meiotic chromosomes. Mitotic chromosomes were used to determine the chromosomal localization of telomeric sequences using the (TTAGGG)n probe. Meiotic chromosomes (pachytene stage) were used to validate our computational predictions for the selected high-copy-number satDNA families, LfoSat004 and LfoSat025. The slides were previously pretreated with RNase A (100 μg/mL in 2× SSC, 1 h at 37 °C) to remove RNA and subsequently treated with pepsin (0.005% in 10 mM HCl, 10 min at 37 °C) to improve probe accessibility. After, they were post-fixed in 1% paraformaldehyde, and dehydrated in an ethanol series (70% 2 min, 90% 2 min, 100% 4 min). The chromosomal denaturation was made in 70% formamide/2× SSC at 72 °C for 3 min. The FISH experiments were performed under high-stringency conditions. Labeled probes were denatured at 75 °C for 10 min prior to being applied to the slides. The hybridization was performed overnight at 37 °C in a humid chamber hybridization. Post-hybridization washes were performed in 2× SSC at 42 °C, followed by detection using an anti-digoxigenin–rhodamine antibody (Roche Diagnostics, Germany; 1:200 dilution) for the satDNA probe, and Alexa Fluor 488 (Invitrogen; 1:200 dilution) for the telomeric probe, according to the manufacturer’s instructions. Chromosomes were counterstained with DAPI and mounted in antifade medium.

## 3 Results

### 3.1 Mitotic and meiotic characterization

**FIGURE 1:**
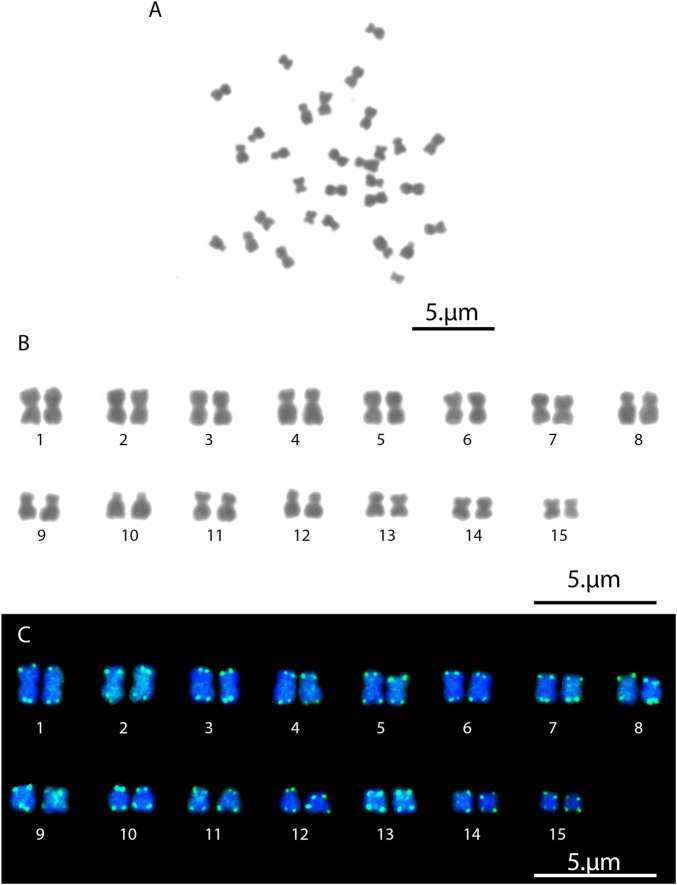
Mitotic chromosomes of Golden Mussel (*Limnoperna fortunei*). **(A)** Representative metaphase spread. **(B)** Karyotype of 2n = 30 chromosomes. **(C)** Karyotype after fluorescence *in situ* hybridization (FISH), showing the telomeric (TTAGGG)_8_ sequences (green signals) on DAPI-stained chromosomes (blue).

**FIGURE 2:**
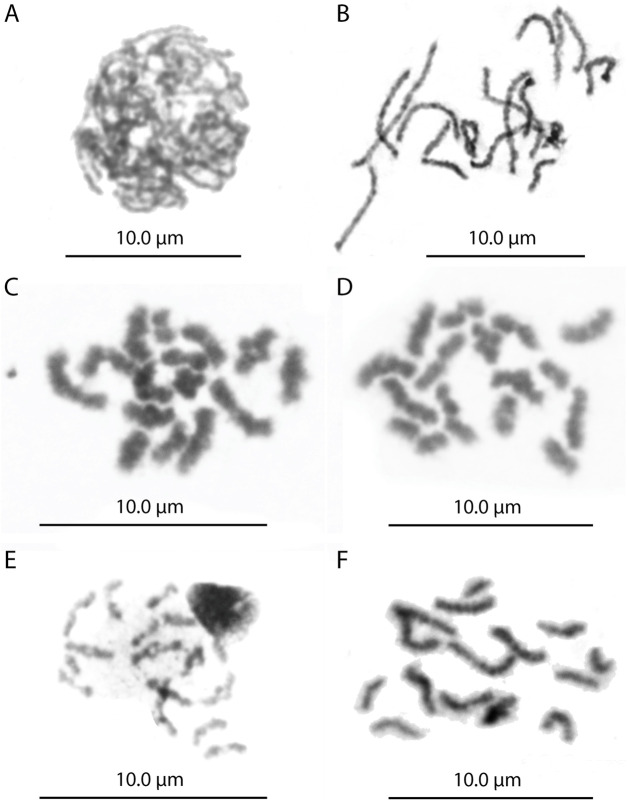
Meiotic cells of female the *Limnoperna fortunei*. **(A)** Zygotene. **(B)** Pachytene with 15 bivalents. **(C)** Diplotene. **(D)** Diakinesis. **(E)** Metaphase I with 15 bivalents. **(F)** Metaphase II with 15 chromosomes.

**FIGURE 3:**
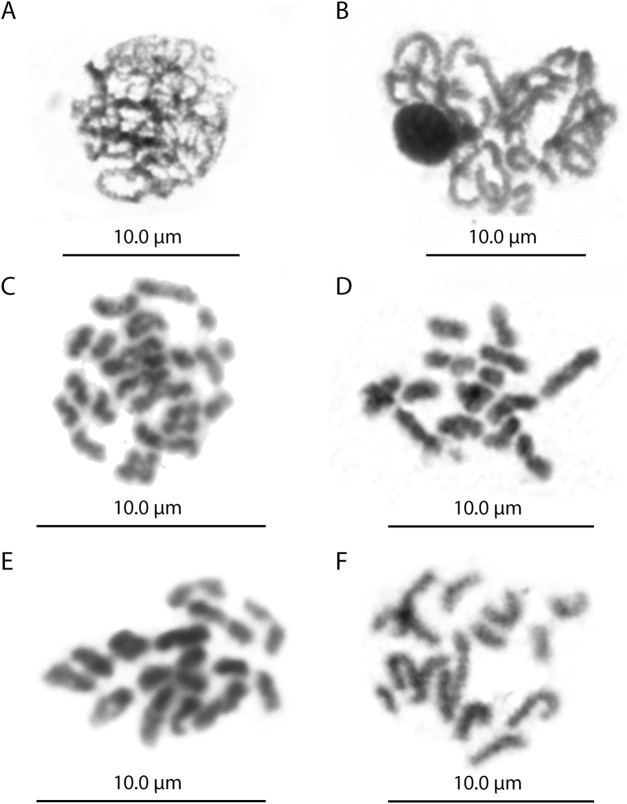
Meiotic cells of male the *Limnoperna fortunei*. **(A)** Zygotene. **(B)** Pachytene with bivalents in bouquet configuration. **(C)** Diplotene. **(D)** Diakinesis. **(E)** Metaphase I with 15 bivalents. **(F)** Metaphase II with 15 chromosomes.

The *L. fortunei* exhibits a diploid chromosome number of 2n = 30 with karyotype formulae (KF) = 20m + 8sm + 2st (Figures 1A,B). FISH with the telomeric probe (TTAGGG)n revealed strong signals at the terminal regions of all mitotic chromosomes (Figure 1C). In addition to the expected terminal signals, dispersed signals were also observed in interstitial regions of several chromosome pairs. Notably, these interstitial signals showed consistent patterns among homologous chromosomes, suggesting they represent genuine (TTAGGG)n repeats rather than background noise. To investigate this further, we performed a comprehensive *in silico* search for tandem arrays of the (TTAGGG)n repeat throughout the chromosome-level genome assembly. This analysis did not identify significant interstitial telomeric sequence (ITS) arrays of substantial length, which may be attributed to assembly quality limitations and the frequent collapse of short repetitive sequences during genome assembly. Nevertheless, the consistent FISH signals in interstitial regions indicate that short (TTAGGG)n repeats or fragmented arrays are present at these loci, even if they are not captured in the current genome assembly. Meiotic chromosome analysis in both males and females confirmed the presence of 15 bivalents (Figures 2, 3). Nuclear DNA content estimation revealed values of 1.4 pg for diploid cells and 0.69 pg for haploid cells from gonadal tissues of both sexes.

Comparative meiotic analysis demonstrated an identical pattern of chromosome segregation in males and females (Figures 2, 3), with no evidence of behaviour or pairing that would suggest the presence of heteromorphic sex chromosomes. During the early meiotic stages (leptotene and zygotene), chromatin condensation and the start of homologous pairing were observed, with bivalents appearing as interlocked structures (Figures 2A, 3A).

At pachytene, both males and females showed the presence of 15 individualized and thicker and more individualized filaments, with different sizes, interpreted as bivalent. Several pachytene nuclei exhibited the typical bouquet configuration (Figures 2B, 3B). Diplotene and diakinesis stages also showed 15 bivalents, corresponding to the meiotic formula 2n = 15II, with one or two chiasma per bivalent (Figures 2C,D, 3C,D). In some diplotene cells, premature segregation of the three bivalents has been observed. At metaphase I (Figures 2E, 3E), bivalents were aligned on the meiotic spindle, while metaphase II cells consistently exhibited 15 chromosomes in both males and females (Figures 2F, 3F).

### 3.2 Satellitome characterization

The initial analysis with RepeatExplorer2 identified 129 distinct satDNA families, which were named LfoSat001-129 according to their abundance rankings in the raw sequencing reads. This analysis estimated a total satDNA content of approximately 9.1% of the genome. The top 20 most abundant families identified by RepeatExplorer2 accounted for approximately 6.2% of the genome, with LfoSat001 being the most abundant (0.9%), followed by LfoSat002 (0.7%) and LfoSat003 (0.6%) (Supplementary Table S1). Subsequently, we performed an *in silico* mapping of these 129 families against the chromosome-level reference genome. This second approach successfully mapped 126 families, which collectively represented approximately 5.3% of the assembled genome (Supplementary Table S2). In this mapping-based analysis, the genomic abundances of individual families varied up to approx. 0.42%, with the top 20 families accounting for approximately 3.9% of the genome. The remaining 106 mapped families contributed an additional 1.4%.

**FIGURE 4:**
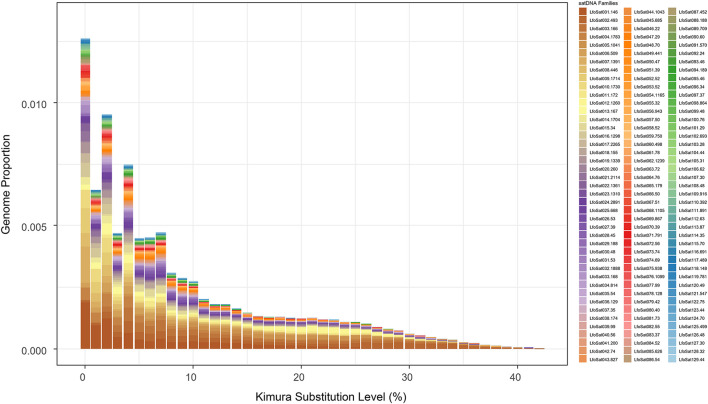
Abundance satDNAs families in Golden Mussel (*Limnoperna fortunei*) relative to the Kimura substitution level. The graph shows the genomic proportion of the satDNA families at different levels of divergence from the consensus sequences. Nomenclature follows RepeatExplorer2 abundance rankings according to Ruiz-Ruano et al. (2016).

**FIGURE 5:**
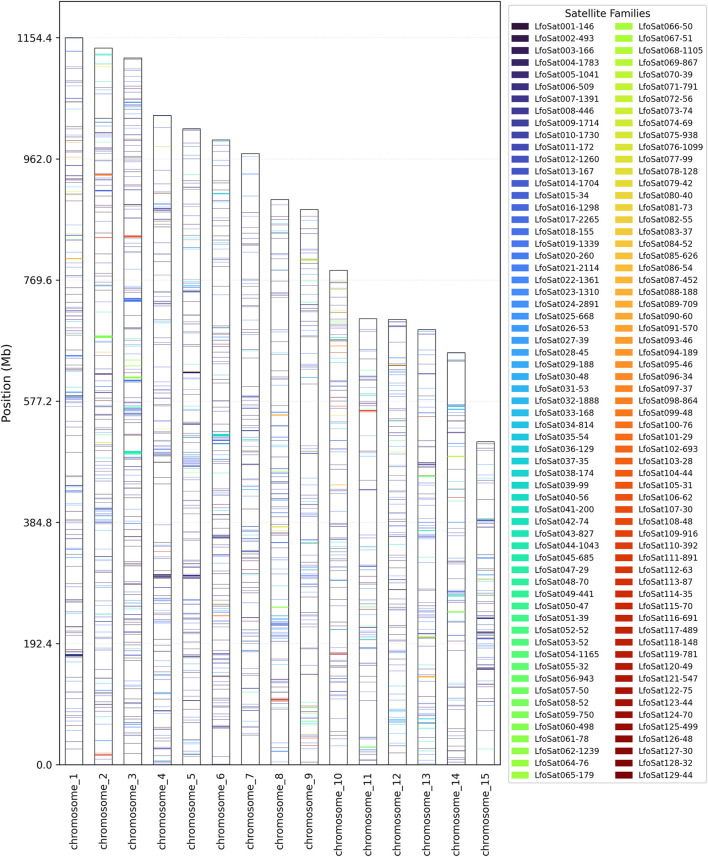
*In silico* mapping of the 126 satDNA families across the 15 chromosomes of Golden Mussel (*Limnoperna fortunei*), based on the reference genome assembly (xbLimFort5.1, NCBI). The distribution pattern highlights the widespread presence of satDNA families throughout the genome, with some exhibiting chromosome-specific enrichment while others are dispersed across multiple chromosomes. Three families (LfoSat046, LfoSat063, and LfoSat092) showed no significant chromosomal mapping, likely due to very low abundance or high sequence divergence below the detection threshold (e-value ≤ 1e-05).

Most families in both analyses are concentrated at low divergence levels (0%–5% Kimura substitutions) (Figure 4 and Supplementary Figure S1, for the top 30 most abundant families.). Three families (LfoSat046, LfoSat063, LfoSat092) were not detected in the chromosomal assembly, likely due to assembly gaps or collapse of highly repetitive regions during genome assembly, despite being more abundant than some chromosome-specific families (Figure 5).

### 3.3 *In silico* and *in situ* chromosome distribution of satDNAs

Chromosome-specific analysis of all 126 mapped families (Figure 5) revealed 32 families (25.4%) showing exclusive chromosomal localization, distributed across 10 of the 15 chromosomes (Supplementary Table S2). The most abundant chromosome-specific families include LfoSat030 (chromosome 12, 0.0236%), LfoSat059 (chromosome 2, 0.0232%), LfoSat119 (chromosome 8, 0.0174%), and LfoSat078 (chromosome 10, 0.0167%). While these families individually represent lower genomic abundances compared to multi-chromosomal families, they represent potential candidates for chromosome-specific markers, pending experimental validation through FISH or other cytogenetic approaches in future studies.

**FIGURE 6:**
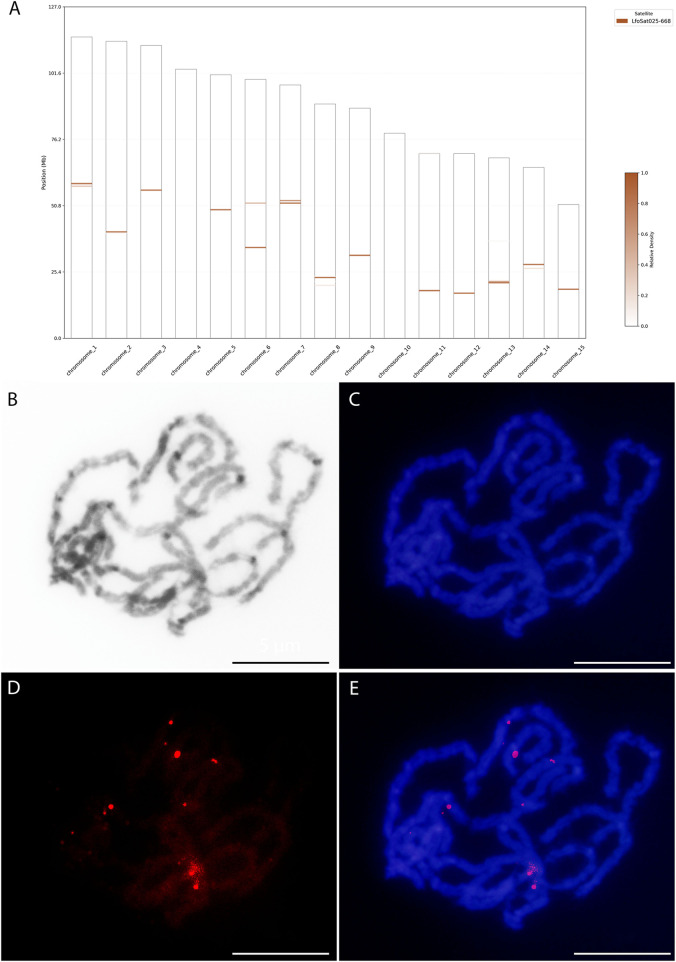
*In silico* and *in situ* chromosomal mapping of the satDNA family LfoSat025 in the *Limnoperna fortunei*. **(A)** *In silico* distribution of LfoSat025 across the 15 chromosomes; the intensity of the color shading corresponds to the relative density of the satDNA sequences according to the scale on the right. **(B)** Pachytenic bivalents with Giemsa. **(C)** counterstained with DAPI (blue). **(D)** FISH signals of the LfoSat025 probe (red), revealing multiple hybridization sites. **(E)** Merged image of DAPI and FISH signals, showing the bivalent localization of LfoSat025. Scale bars = 5 µm.

**FIGURE 7:**
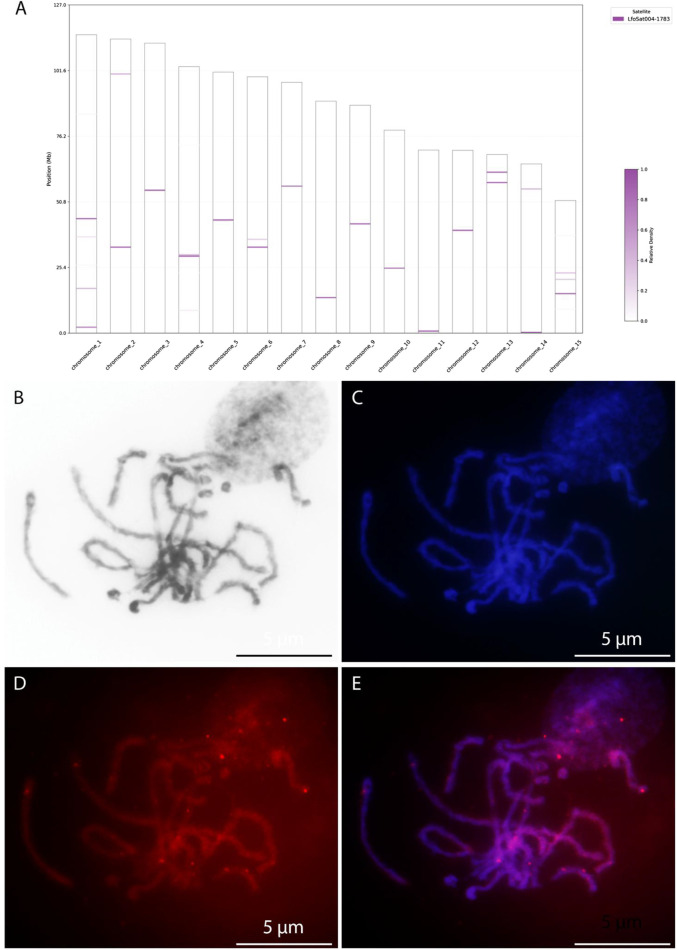
*In silico* and *in situ* chromosomal mapping of the satDNA family LfoSat004 in the *Limnoperna fortunei*. **(A)** *In silico* distribution of LfoSat004 across the 15 chromosomes; the intensity of the color shading corresponds to the relative density of the satDNA sequences according to the scale on the right. **(B)** Pachytenic bivalents with Giemsa. **(C)** counterstained with DAPI (blue). **(D)** FISH signals of the LfoSat004 probe (red), revealing multiple hybridization sites. **(E)** Merged image of DAPI and FISH signals, showing the bivalent localization of LfoSat025. Scale bars = 5 µm.

**FIGURE 8:**
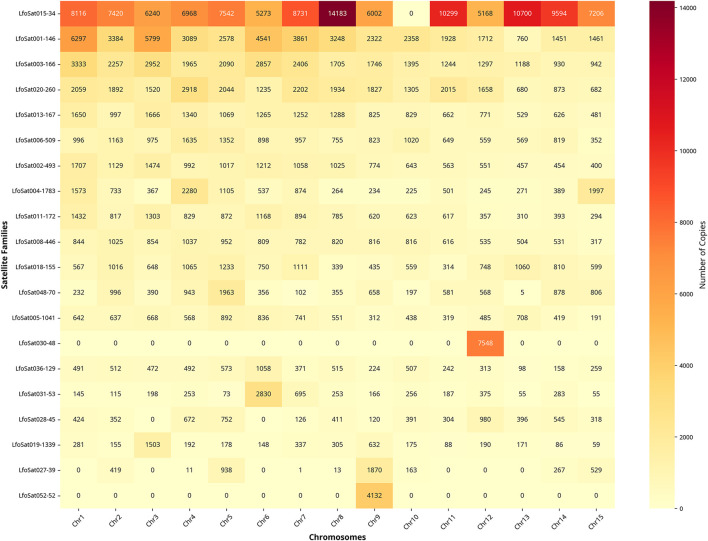
Chromosomal distribution of the top 20 satDNA families ranked by total copy number according to *in silico* analysis of *Limnoperna fortunei*. Color intensity represents copy number per chromosome (scale 0–14,183). Families ordered by decreasing total copy number, which differs from nomenclature ranking based on RepeatExplorer2 abundance estimates. BLAST analysis: e-value ≤ 1e-05, minimum identity 80%, minimum overlap 80%.

Among the 126 mapped families, LfoSat025 and LfoSat004 were selected for FISH validation due to their distinctive distribution patterns. LfoSat025 showed local enrichment across 13 chromosomes (absent on chromosomes 4 and 10), with a pattern suggesting pericentromeric localization (Figure 6). LfoSat004, one of the most abundant families (0.29% of the genome, 11,595 copies), exhibited local enrichment across all 15 chromosomes, demonstrating that pan-chromosomal distribution can be associated with regional specificity (Figure 7). Fluorescence *in situ* hybridization confirmed the *in silico* predictions for both families. LfoSat025 (Figure 6) showed preferential localization in the pericentromeric regions of pachytene bivalents (Figures 6B–E). LfoSat004 (Figure 7) displayed multiple localized signals across pachytene bivalents, with regional accumulation (Figures 7B–E). Furthermore, the *in silico* analysis also allowed evaluation of the copy number of each satDNA family, with the 20 most abundant highlighted in Figure 8.

The satellite LfoSat015-34 was the most abundant element, with 113,442 copies distributed across 14 of the 15 chromosomes analyzed (absent only on chromosome 10), suggesting a significant role in genomic structure unrelated to centromeric or telomeric regions (Figure 8). This sequence showed no matches in public databases (NCBI, Repbase), indicating it is likely species-specific with a function yet to be determined. The diversity of satDNA families per chromosome varied considerably, with chromosome 2 presenting the highest richness (65 families), while chromosome 1 concentrated the largest absolute number of copies (36,450).

## 4 Discussion

This study integrates cytogenetic validation through FISH with genome-wide bioinformatic analysis to characterize the chromosomal distribution of satDNAs in the *L. fortunei* genome. Our results reveal that the species possesses a diversified and widely distributed repertoire of these repetitive elements in its genome and chromosomes. The apparent paradox between the adaptive success of an invasive species and the reduction in genetic variability caused by population bottlenecks, as observed in the case of this Asian mussel in South American environments, can now be reinterpreted in light of karyotypic microevolution.

### 4.1 Diversity and evolutionary dynamics of the satellitome

The identification of 129 distinct satDNA families in *L. fortunei* reveals a rich and complex satellitome, contributing to the growing body of knowledge on repetitive elements in bivalve mollusks. This diversity is comparable to that found in other well-studied bivalves, where satellitomes can be highly dynamic and structurally diverse (e.g., Tunjić-Cvitanić et al., 2021).

The relatively moderate satDNA content in *L. fortunei* (approx. 9.1% of the genome) falls within the range reported for other bivalve species, such as the Pacific oyster *Crassostrea gigas* (5%–8%), but contrasts with some terrestrial mollusks where satellitomes can exceed 20% of the genome, suggesting potential ecological or physiological constraints on repetitive DNA accumulation in aquatic bivalves. Similarly, in the hard clam *Mercenaria mercenaria* (Veneridae), satellite DNA elements were found to represent approximately 10% of the genome (Farhat et al., 2022).

These abundance estimates, however, must be interpreted in light of important methodological considerations. Both abundance values and divergence estimates differ significantly between the two methods employed in this study. RepeatExplorer2 provides genome-wide estimates based on read clustering, which is sensitive to all repetitive DNA in the raw data, including highly divergent and ancient copies. In contrast, *in silico* mapping quantifies discrete copies on the assembled chromosomes using BLAST with stringent parameters (e-value ≤ 1e-05, minimum identity 80%, minimum overlap 80%). This approach is susceptible to two main biases: (i) underestimation of abundance due to assembly gaps or the collapse of highly repetitive regions in the reference genome, and (ii) underestimation of divergence due to mapping bias, as BLAST preferentially maps more conserved copies while excluding highly divergent variants that fail to meet the identity threshold. Consequently, the *in silico* approach captures a younger, more homogeneous subset of each satDNA family, resulting in systematically lower divergence values (mean: 3.98% vs. 7.21% in RepeatExplorer2) and altered abundance rankings. These methodological differences explain why some families with higher nomenclature numbers (lower abundance in RepeatExplorer2) appear among the most abundant in the chromosomal mapping analysis, and why divergence values are consistently lower in the *in silico* analysis (Supplementary Tables S1,S2).

LfoSat025 showed localized accumulation in specific regions of pachytene bivalents, with a pattern consistent with pericentromeric localization. While we did not functionally prove their role, this pattern suggests that LfoSat025 could be involved in the organization of pericentromeric heterochromatin. About this, it is crucial to recognize that this is not the only possible distribution for satDNA. Recent studies have increasingly challenged the paradigm that satDNAs are exclusively confined to heterochromatic regions. For instance, a substantial portion of the satellitome in *C*. *gigas*, is highly scattered across the genome, suggesting potential roles in gene regulation or other functions beyond structural heterochromatin (Tunjić-Cvitanić et al., 2021). Similarly, a study in Neuropteran insects also revealed that abundant satDNAs acmulated in euchromatin, providing further evidence that satDNA distribution patterns are highly species-specific and not universally linked to heterochromatin (Cabral-de-Mello et al., 2025).

Our *in silico* mapping also reveals families in *L. fortunei* with pan-chromosomal distribution, such as LfoSat004, which is present on all 15 chromosomes with localized regional enrichment. The chromatin context and potential functional significance of these pan-chromosomal families remain to be determined through additional approaches such as ChIP-seq or chromatin immunoprecipitation experiments.

The divergence profile reveals distinct evolutionary patterns within the *L. fortunei* satellitome. The predominance of low-divergence variants (0%–5% Kimura substitutions) across most families indicates recent amplification events, a pattern consistent with the “molecular drive” model of satDNA evolution (Dover, 1982; Ruiz-Ruano et al., 2016; João Da Silva et al., 2023). This recent expansion could be associated with population bottlenecks during South American colonization or adaptive responses to novel environments (Lower et al., 2018).

The presence of higher-divergence variants in the same families suggests either: (1) multiple amplification events separated in time, reflecting cycles of amplification and degeneration (Camacho et al., 2022); (2) differential evolutionary rates within satellite arrays, where some regions evolve faster than others (Vittorazzi et al., 2014); or (3) remnants of ancestral sequences undergoing gradual decay (Camacho et al., 2022). This complex pattern, with both recent and older variants coexisting, differs from some bivalve species where ancient, highly divergent satellite families dominate the satellitome, such as the BIV160 satDNA found across multiple bivalve clades (Plohl et al., 2010; Šatović et al., 2018) This suggests a more dynamic and recent evolutionary history of repetitive elements in *L. fortunei* compared to species with more ancient satDNA profiles.

Notably, some satellite families demonstrated pan-chromosomal distribution, being present in all 15 chromosomes of the species’ standard complement, indicating possible functional and/or structural importance. The significant variation in abundance and diversity between chromosomes suggests specific patterns of genomic organization, possibly related to differential evolution of repetitive elements or chromosome-specific selective pressures. The asymmetric abundance distribution observed, where few families dominate the satellite landscape, reflects the stochastic nature of satDNA amplification and decay processes. This pattern suggests that satDNA families undergo cycles of expansion and contraction over evolutionary time, with successful families expanding rapidly while others diminish through mutational decay or deletion events (Camacho et al., 2022).

The size of repetitive units, ranging from 22 to 2,891 bp, encompasses a wide range typical of satDNA in eukaryotes (Garrido-Ramos, 2017; Thakur et al., 2021) The satDNAs with shorter monomer sizes may represent ancestral elements that underwent deletions or fragmentation over evolutionary time, while larger ones may have resulted from internal duplications or fusions of smaller units (Camacho et al., 2022) This size diversity suggests multiple mechanisms of satDNA origin and evolution in *L. fortunei*, including unequal crossing-over, replication slippage, transposon-mediated duplications, and *de novo* emergence from tandem duplications of simple sequences (Plohl et al., 2008; Garrido-Ramos, 2017).

The low divergence observed in most *L. fortunei* satellite families contrasts with patterns found in some bivalve species where ancient satDNA with high divergence is common, such as in certain Veneridae species where satDNA subfamilies can differ by up to 11% (Plohl and Cornudella, 1996; Plohl et al., 2010). However, it aligns with patterns observed in other Mytilidae, where satDNAs show greater conservation among closely related species (Martínez-Lage et al., 2002; Martínez-Lage et al., 2005), suggesting family-specific evolutionary dynamics of satDNA (García-Souto et al., 2015; Šatović et al., 2018).

### 4.2 Chromosomal organization and functional implications

The karyotype and diploid number of *L. fortunei* 2n = 30 observed in this work is consistent with other studies and species of the Mytilidae family (now assigned to the family Modiolidae, following a recent taxonomic revision by Tan and Tan, 2024), in which diploid numbers vary from 2n = 22 to 2n = 32 chromosomes (Thiriot-Quievreux, 2002). There is extensive literature describing patterns of satDNA distribution in bivalves and other taxa (Šatović et al., 2018; Cabral-de-Mello et al., 2021; Šatović-Vukšić and Plohl, 2023). The relatively uniform distribution of satDNAs across the 15 chromosomes in *L. fortunei*, revealed by *in silico* mapping, is comparable to the scattered organization observed in *C. gigas* (Tunjić-Cvitanić et al., 2021) and reflects the diversity of satDNA distribution patterns documented across bivalve species (Šatović et al., 2018). While some species show highly localized satDNA distributions restricted to specific chromosomes or regions (Biscotti et al., 2007; Petrović et al., 2009), others, like *L. fortunei*, exhibit more widespread patterns across multiple chromosomes (Martínez-Lage et al., 2002; Vojvoda Zeljko et al., 2020).

The identification of 32 chromosome-specific satDNA families (25.4% of mapped families) in *L. fortunei* provides a valuable resource for chromosome identification and cytogenetic studies (Wei et al., 2020; Marková et al., 2025). While these families tend to have lower individual abundances compared to the highly abundant multi-chromosomal families, their chromosome specificity makes them particularly useful as molecular cytogenetic markers, a principle well-established in bivalve cytogenetics (Clabby et al., 1996; Thiriot-Quievreux, 2002). This proportion of chromosome-specific families is comparable to patterns observed in other bivalve species (Šatović et al., 2018) and suggests that satDNA evolution in *L. fortunei* involves both chromosome-specific amplification events and genome-wide dispersal mechanisms (Thakur et al., 2021).

The presence of low-copy satDNA occurrences (1-5 copies per chromosome, represented by light yellow in Figure 8) raises intriguing questions about satDNA evolutionary dynamics and genome organization in *L. fortunei*. These sporadic distributions may reflect several evolutionary processes that have shaped the current satellitome architecture. First, they could represent evolutionary remnants of once-larger satellite arrays that have been progressively disrupted through transposable element insertions, chromosomal rearrangements, or gradual sequence divergence beyond recognition thresholds. Such degradation processes have been documented in other species where satDNA families undergo cycles of amplification and subsequent decay (Plohl et al., 2008; Garrido-Ramos, 2017).

Alternatively, these low-copy occurrences might indicate recent transposition or recombination events that have dispersed satellite sequences to new chromosomal locations, potentially representing early stages of satellite colonization of previously unoccupied genomic regions. This interpretation is supported by studies in *Drosophila* and other organisms where satDNA mobility contributes to genome evolution (Jagannathan et al., 2018). However, it cannot be excluded that some of these occurrences represent assembly artifacts inherent to the challenges of accurately assembling highly repetitive genomic regions, particularly in complex satDNA landscapes. To distinguish between biologically meaningful satellite distributions and potential background noise, some authors assume a conservative threshold of 10 copies per chromosome (Satović et al., 2016). This approach allows focus on satellite families with substantial chromosomal presence while acknowledging that low-copy occurrences may harbor important evolutionary information worthy of future investigation using more sensitive detection methods or experimental validation approaches.

The LfoSat025 family (Figure 6) showed a clear accumulation in condensed, DAPI-positive regions of pachytene bivalents, which is consistent with a pericentromeric localization. While we did not functionally prove their role, this pattern suggests that LfoSat025 could be involved in the organization of pericentromeric heterochromatin. This hypothesis is in line with current models where pericentromeric satDNAs are considered important for maintaining centromere integrity and ensuring proper chromosome segregation, often through the formation of specialized chromatin domains (Brändle et al., 2022; Ugarković et al., 2022). In contrast, LfoSat004 (Figure 7) exhibited localized enrichment between the bivalents without clear pericentromeric restriction, demonstrating that satDNAs in *L. fortunei* can show diverse distribution patterns. This diversity, with some families showing chromosome-specific pericentromeric localization and others showing pan-chromosomal regional accumulation, reflects the complex organizational landscape of the *L. fortunei* satellitome.

### 4.3 Meiotic behavior and reproductive biology

Meiotic analysis revealed normal chromosomal behavior with regular pairing and segregation, confirming the presence of 15 bivalents (2n = 15II) in both males and females. This pattern indicates genomic stability and normal meiotic processes, suggesting that the satDNA content, while significant, does not grossly disrupt chromosomal behavior during gametogenesis. It is important to note, however, that the ∼9.1% of the genome represented by satDNAs in *L. fortunei* is moderate compared to many other species where this fraction can exceed 50% and is often distributed across all chromosomes (Garrido-Ramos, 2017). The presence of normal meiotic behavior is consistent with observations across diverse taxa, where meiosis functions effectively regardless of satellitome size, indicating that satDNA content *per se* does not necessarily disrupt chromosomal behavior during gametogenesis.

Recent studies have shown that satDNAs are not merely structural components but can be actively involved in cellular responses to environmental stress. For example, in the beetle *Tribolium castaneum*, the major satDNA (TCAST1) is transcribed in response to heat stress, and its transcripts are involved in modulating the expression of stress-related genes (Feliciello et al., 2015; Sermek et al., 2020). The controlled abundance and organization of the *L. fortunei* satellitome might represent an evolutionary trade-off, maintaining genomic stability while allowing for a degree of plasticity in cellular stress responses (Fonseca-Carvalho et al., 2024).

The absence of sex chromosome differentiation confirms previous cytogenetic observations and provides insights into the reproductive biology of this economically important species (Reis et al., 2023). *L. fortunei* is predominantly gonochoristic (dioecious), with approximately equal ratios of males and females and external fertilization. However, occasional hermaphroditism has been documented in less than 0.6% of populations, with some studies reporting higher frequencies during early invasion periods (Fernandes de Oliveira et al., 2025). This reproductive mode, combined with the absence of sex-specific chromosomal markers, suggests that sex determination in *L. fortunei* likely involves environmental factors or subtle genetic mechanisms not detectable through conventional karyotyping.

The bouquet configuration observed during pachytene is a conserved phenomenon in many eukaryotes and is associated with efficient homologous pairing and recombination (Scherthan, 2007). The presence of this configuration in *L. fortunei* suggests conserved meiotic mechanisms and may contribute to the observed genomic stability. Bouquet formation may also facilitate the resolution of chromosomal entanglements caused by repetitive sequences (Scherthan, 2001). The observation of early segregation in some bivalents during diplotene may indicate occasional meiotic irregularities, but its low frequency suggests it does not significantly compromise the species’ reproductive viability. This meiotic stability may contribute to the reproductive success of *L. fortunei* in diverse environments. Furthermore, the presence of one chiasma per bivalent is consistent with the minimum necessary to ensure adequate pairing and segregation during meiosis I (Page and Hawley, 2003). This conservative recombination pattern may reflect an evolutionary strategy for maintaining genomic integrity in the face of the abundant repetitive fraction of the observed genome.

### 4.4 Satellitome and adaptive potential in invasive species

The relationship between satellitome characteristics and invasive success represents an emerging area of research in invasion biology. While direct causal relationships are difficult to establish, several aspects of the *L. fortunei* satellitome may contribute to the species’ adaptive potential. The high diversity of satDNA families (129) and evidence of recent amplification events suggest an active and dynamic repetitive DNA landscape that could facilitate genomic plasticity.

Repetitive sequences, including satDNA, can contribute to adaptive evolution through several mechanisms: (1) they can promote chromosomal rearrangements that generate genetic variability (George and Alani, 2012; Liao et al., 2023), (2) they can influence gene regulation through position effects or epigenetic modifications (Fedoroff, 2012; Ferreira et al., 2019), and (3) they can serve as substrates for the evolution of new genes or regulatory elements (Stewart and Rogers, 2019; Snowbarger et al., 2024). The recent amplification of multiple satDNA families in *L. fortunei*, evidenced by low divergence levels (João Da Silva et al., 2023; Pita et al., 2025), may be related to population bottleneck events during South American colonization (Yao et al., 2020) or to specific selective pressures encountered in new environments (Lavergne and Molofsky, 2007; Mounger et al., 2021).

Studies have demonstrated that repetitive elements can be activated during stress conditions or colonization events in various organisms (Casacuberta and González, 2013). While we cannot directly demonstrate such activation in *L. fortunei*, the patterns of recent satDNA amplification observed in our study are consistent with genomic responses to environmental challenges. The dispersed distribution of satDNAs across chromosomes may also contribute to genome-wide regulatory effects that could influence adaptive responses.

The identification of chromosome-specific satDNA markers in *L. fortunei* provides valuable tools for population genetic studies that could help elucidate the role of genomic factors in invasion success. These markers could be used to trace invasion routes, identify source populations, and monitor genetic changes during establishment in new environments.

## 5 Conclusion

This study provides the first comprehensive characterization of the satDNA landscape in the invasive golden mussel *Limnoperna fortunei*, revealing a remarkably diverse satelitome comprising 129 distinct families. The predominance of recently amplified satellite sequences indicates ongoing satDNA turnover in the *L. fortunei* genome. The integrated *in silico* and *in situ* approaches provide a framework for future genomic and cytogenetic studies of repetitive DNA organization in this invasive species. Specifically, our findings contribute to the understanding of repetitive DNA in successful aquatic invaders by: (i) delivering the first comprehensive satellitome for this ecologically critical species; (ii) establishing a new set of molecular markers for population and invasion-route studies; and (iii) revealing patterns of recent satDNA amplification that suggest dynamic genomic responses to colonization events.

## Data Availability

Raw sequencing data have been deposited in the NCBI Sequence Read Archive (SRA) under BioProject accession number PRJNA1389674.

## References

[B1] Almeida M. C. Zacaro A. A. Cella D. M. (2000). Cytogenetic analysis of epicauta atomavia (Meloidae) and palembus dermestoides (Tenebrionidae) with xyp sex determination system using standard staining, C-Bands, NOR and synaptonemal complex microspreading techniques. Hereditas 133, 147–157. 10.1111/j.1601-5223.2000.00147.x 11338427

[B2] Biscotti M. A. Canapa A. Olmo E. Barucca M. Teo C. H. Schwarzacher T. (2007). Repetitive DNA, molecular cytogenetics and genome organization in the king scallop (Pecten maximus). Gene 406, 91–98. 10.1016/j.gene.2007.06.027 17706376

[B3] Biscotti M. A. Canapa A. Forconi M. Olmo E. Barucca M. (2015). Transcription of tandemly repetitive DNA: functional roles. Chromosome Res. 23, 463–477. 10.1007/s10577-015-9494-4 26403245

[B4] Boltovskoy D. (2015). Distribution and colonization of limnoperna fortunei: special traits of an odd mussel. In: Limnoperna fortunei. Cham: Springer International Publishing, 301–311. 10.1007/978-3-319-13494-9_16

[B5] Bourque G. Burns K. H. Gehring M. Gorbunova V. Seluanov A. Hammell M. (2018). Ten things you should know about transposable elements. Genome Biol. 19, 199. 10.1186/s13059-018-1577-z 30454069 PMC6240941

[B6] Brändle F. Frühbauer B. Jagannathan M. (2022). Principles and functions of pericentromeric satellite DNA clustering into chromocenters. Semin. Cell Dev. Biol. 128, 26–39. 10.1016/j.semcdb.2022.02.005 35144860

[B7] Cabral-de-Mello D. C. Zrzavá M. Kubíčková S. Rendón P. Marec F. (2021). The role of satellite DNAs in genome architecture and sex chromosome evolution in crambidae moths. Front. Genet. 12, 661417. 10.3389/fgene.2021.661417 33859676 PMC8042265

[B8] Cabral-de-Mello D. C. Gasparotto A. E. Rico-Porras J. M. Ferretti A. B. S. M. Mora P. Alves-Gomes R. T. (2025). First insights into the satellitomes and new evidence for the absence of canonical insect telomere in the Neuroptera order. Genome 68, 1–12. 10.1139/gen-2025-0018 40446331

[B9] Camacho J. P. M. Cabrero J. López-León M. D. Martín-Peciña M. Perfectti F. Garrido-Ramos M. A. (2022). Satellitome comparison of two oedipodine grasshoppers highlights the contingent nature of satellite DNA evolution. BMC Biol. 20, 36. 10.1186/s12915-021-01216-9 35130900 PMC8822648

[B10] Casacuberta E. González J. (2013). The impact of transposable elements in environmental adaptation. Mol. Ecol. 22, 1503–1517. 10.1111/mec.12170 23293987

[B11] Chen Z. Baeza J. A. Chen C. Gonzalez M. T. González V. L. Greve C. (2025). A genome-based phylogeny for mollusca is concordant with fossils and morphology. Sci. (1979) 387, 1001–1007. 10.1126/science.ads0215 40014700

[B12] Clabby C. Goswami U. Flavin F. Wilkins N. P. Houghton J. A. Powell R. (1996). Cloning, characterization and chromosomal location of a satellite DNA from the Pacific oyster, Crassostrea gigas. Gene 168, 205–209. 10.1016/0378-1119(95)00749-0 8654945

[B13] Darrigran G. Damborenea C. (2011). Ecosystem engineering impact of *Limnoperna fortunei* in South America. Zool. Sci. 28, 1–7. 10.2108/zsj.28.1 21186940

[B14] Darrigran G. Pastorino G. (1995). The recent introduction of a freshwater asiatic bivalve, Limnoperna fortunei (Mytilidae) into South America. Veliger 38, 171–175.

[B15] Dei Tos C. Quagio Grassiotto I. Mazzoni T. S. (2016). Cellular development of the germinal epithelium during the gametogenic cycle of the golden mussel Limnoperna fortunei (Bivalvia: mytilidae). Rev. Biol. Trop. 64, 521–536. 10.15517/rbt.v64i2.18837 29451752

[B16] Dover G. (1982). Molecular drive: a cohesive mode of species evolution. Nature 299, 111–117. 10.1038/299111a0 7110332

[B67] Dunker W. (1857). Mytilidae novae collectionis Cumingianae. Proceedings of the Zoological Society of London 25, 358–366.

[B17] Ebied A.-B. M. Aly F. M. (2004). Cytogenetic studies on metaphase chromosomes of six bivalve species of families Mytilidae and Veneridae (Nucinelloidea, Mollusca). Cytol. (Tokyo) 69, 261–273. 10.1508/cytologia.69.261

[B18] Farhat S. Bonnivard E. Pales Espinosa E. Tanguy A. Boutet I. Guiglielmoni N. (2022). Comparative analysis of the mercenaria mercenaria genome provides insights into the diversity of transposable elements and immune molecules in bivalve mollusks. BMC Genomics 23, 192. 10.1186/s12864-021-08262-1 35260071 PMC8905726

[B19] Fedoroff N. V. (2012). Transposable elements, epigenetics, and genome evolution. Sci. (1979) 338, 758–767. 10.1126/science.338.6108.758 23145453

[B20] Feliciello I. Akrap I. Ugarković Đ. (2015). Satellite DNA modulates gene expression in the beetle *Tribolium castaneum* after heat stress. PLoS Genet. 11, e1005466. 10.1371/journal.pgen.1005466 26275223 PMC4537270

[B21] Fernandes de Oliveira P. R. F. Rodrigues C. C. Teixeira de Sabóia-Morais S. M. Grano-Maldonado M. I. Rocha T. L. (2025). Reproductive strategies of the golden mussel (Limnoperna fortunei) during the initial invasion in the Brazilian cerrado. Invertebr. Biol. 144. 10.71161/ivb.144.2.2024.00005

[B22] Ferreira D. Escudeiro A. Adega F. Chaves R. (2019). DNA methylation patterns of a satellite non-coding sequence – FA-SAT in cancer cells: its expression cannot be explained solely by DNA methylation. Front. Genet. 10, 101. 10.3389/fgene.2019.00101 30809250 PMC6379292

[B23] Fonseca-Carvalho M. Veríssimo G. Lopes M. Ferreira D. Louzada S. Chaves R. (2024). Answering the cell stress call: satellite non-coding transcription as a response mechanism. Biomolecules 14, 124. 10.3390/biom14010124 38254724 PMC10813801

[B68] García-Souto D. Pérez-García C. Morán P. Pasantes J. J. (2015). Divergent evolutionary behavior of H3 histone gene and rDNA clusters in venerid clams. Mol. Cytogenet. 8 (1), 40. 10.1186/s13039-015-0150-7 26106449 PMC4477615

[B24] Garrido-Ramos M. (2017). Satellite DNA: an evolving topic. Genes (Basel) 8, 230. 10.3390/genes8090230 28926993 PMC5615363

[B25] George C. M. Alani E. (2012). Multiple cellular mechanisms prevent chromosomal rearrangements involving repetitive DNA. Crit. Rev. Biochem. Mol. Biol. 47, 297–313. 10.3109/10409238.2012.675644 22494239 PMC3337352

[B26] Ieyama H. (1996). Chromosomes and nuclear DNA contents of limnoperna in Japan (bivalvia: mytilidae). Malacological Society Jpn. 55, 65–68.

[B27] Jagannathan M. Cummings R. Yamashita Y. M. (2018). A conserved function for pericentromeric satellite DNA. Elife 7, e34122. 10.7554/eLife.34122 29578410 PMC5957525

[B28] João Da Silva M. Gazoni T. Haddad C. F. B. Parise-Maltempi P. P. (2023). Analysis in Proceratophrys boiei genome illuminates the satellite DNA content in a frog from the Brazilian Atlantic forest. Front. Genet. 14, 1101397. 10.3389/fgene.2023.1101397 37065500 PMC10095563

[B29] Lavergne S. Molofsky J. (2007). Increased genetic variation and evolutionary potential drive the success of an invasive grass. Proc. Natl. Acad. Sci. 104, 3883–3888. 10.1073/pnas.0607324104 17360447 PMC1805698

[B30] Liao X. Zhu W. Zhou J. Li H. Xu X. Zhang B. (2023). Repetitive DNA sequence detection and its role in the human genome. Commun. Biol. 6, 954. 10.1038/s42003-023-05322-y 37726397 PMC10509279

[B31] Lower S. S. McGurk M. P. Clark A. G. Barbash D. A. (2018). Satellite DNA evolution: old ideas, new approaches. Curr. Opin. Genet. Dev. 49, 70–78. 10.1016/j.gde.2018.03.003 29579574 PMC5975084

[B32] Marková A. Orosová M. Mora P. Benovics M. Lorite P. (2025). The first insight into acanthocephalus (Palaeacanthocephala) satellitome: species-specific satellites as potential cytogenetic markers. Sci. Rep. 15, 2945. 10.1038/s41598-025-85728-2 39849044 PMC11758010

[B33] Martínez-Lage A. Rodríguez F. González-Tizón A. Prats E. Cornudella L. Méndez J. (2002). Comparative analysis of different satellite DNAs in four *mytilus* species. Genome 45, 922–929. 10.1139/g02-056 12416625

[B34] Martínez-Lage A. Rodríguez-Fariña F. González-Tizón A. Méndez J. (2005). Origin and evolution of *Mytilus* mussel satellite DNAs. Genome 48, 247–256. 10.1139/g04-115 15838547

[B35] Mounger J. Ainouche M. L. Bossdorf O. Cavé-Radet A. Li B. Parepa M. (2021). Epigenetics and the success of invasive plants. Philosophical Trans. R. Soc. B Biol. Sci. 376, 20200117. 10.1098/rstb.2020.0117 33866809 PMC8059582

[B36] Novák P. Neumann P. Pech J. Steinhaisl J. MacAs J. (2013). RepeatExplorer: a Galaxy-based web server for genome-wide characterization of eukaryotic repetitive elements from next-generation sequence reads. Bioinformatics 29, 792–793. 10.1093/bioinformatics/btt054 23376349

[B37] Page S. L. Hawley R. S. (2003). Chromosome choreography: the meiotic ballet. Sci. (1979) 301, 785–789. 10.1126/science.1086605 12907787

[B38] Pastorino G. Darrigran G. Martín S. M. Lunaschi L. (1993). Limnoperna fortunei (Dunker, 1857) (Mytilidae), nuevo bivalvo invasor en aguas del Río de la Plata. Neotrópica 39, 34.

[B39] Petrović V. Pérez-García C. Pasantes J. J. Šatović E. Prats E. Plohl M. (2009). A GC-rich satellite DNA and karyology of the bivalve mollusk *Donax trunculus:* a dominance of GC-rich heterochromatin. Cytogenet Genome Res. 124, 63–71. 10.1159/000200089 19372670

[B40] Pita S. Mora P. Rico‐Porras J. M. Cabral‐de‐Mello D. C. Ruiz‐Ruano F. J. Palomeque T. (2025). A new piece in the repeatome puzzle of triatominae bugs: the analysis of *Triatoma rubrofasciata* reveals the role of satellite DNAs in the karyotypic evolution of distinct lineages. Insect Mol. Biol. 34, 917–928. 10.1111/imb.13013 40576357 PMC12604443

[B41] Plohl M. Cornudella L. (1996). Characterization of a complex satellite DNA in the mollusc donax trunculus: analysis of sequence variations and divergence. Gene 169, 157–164. 10.1016/0378-1119(95)00734-2 8647440

[B42] Plohl M. Luchetti A. Meštrović N. Mantovani B. (2008). Satellite DNAs between selfishness and functionality: structure, genomics and evolution of tandem repeats in centromeric (hetero)chromatin. Gene 409, 72–82. 10.1016/j.gene.2007.11.013 18182173

[B43] Plohl M. Petrović V. Luchetti A. Ricci A. Šatović E. Passamonti M. (2010). Long-term conservation vs high sequence divergence: the case of an extraordinarily old satellite DNA in bivalve mollusks. Hered. (Edinb) 104, 543–551. 10.1038/hdy.2009.141 19844270

[B44] Reis A. C. Amaral D. Americo J. A. Rebelo M. F. Sousa S. M. (2023). Cytogenetic characterization of the golden mussel (Limnoperna fortunei) reveals the absence of sex heteromorphic chromosomes. An Acad Bras Cienc 95, e20201622. 10.1590/0001-3765202320201622 37341265

[B45] Ruiz-Ruano F. J. López-León M. D. Cabrero J. Camacho J. P. M. (2016). High-throughput analysis of the satellitome illuminates satellite DNA evolution. Sci. Rep. 6, 28333. 10.1038/srep28333 27385065 PMC4935994

[B46] Satović E. Vojvoda Zeljko T. Luchetti A. Mantovani B. Plohl M. (2016). Adjacent sequences disclose potential for intra-genomic dispersal of satellite DNA repeats and suggest a complex network with transposable elements. BMC Genomics 17, 997. 10.1186/s12864-016-3347-1 27919246 PMC5139131

[B47] Šatović E. Vojvoda Zeljko T. Plohl M. (2018). Characteristics and evolution of satellite DNA sequences in bivalve mollusks. Eur. Zool. J. 85, 94–103. 10.1080/24750263.2018.1443164

[B48] Šatović-Vukšić E. Plohl M. (2023). Satellite DNAs—From localized to highly dispersed genome components. Genes (Basel) 14, 742. 10.3390/genes14030742 36981013 PMC10048060

[B49] Scherthan H. (2001). A bouquet makes ends meet. Nat. Rev. Mol. Cell Biol. 2, 621–627. 10.1038/35085086 11483995

[B50] Scherthan H. (2007). Telomeres and meiosis in health and disease. Cell. Mol. Life Sci. 64, 117–124. 10.1007/s00018-006-6463-2 17219026 PMC11136286

[B51] Schrader L. Schmitz J. (2019). The impact of transposable elements in adaptive evolution. Mol. Ecol. 28, 1537–1549. 10.1111/mec.14794 30003608

[B52] Sermek A. Feliciello I. Ugarković Đ. (2020). Distinct regulation of the expression of satellite DNAs in the beetle Tribolium castaneum. Int. J. Mol. Sci. 22, 296. 10.3390/ijms22010296 33396654 PMC7796160

[B53] Shatskikh A. S. Kotov A. A. Adashev V. E. Bazylev S. S. Olenina L. V. (2020). Functional significance of satellite DNAs: insights from drosophila. Front. Cell Dev. Biol. 8, 312. 10.3389/fcell.2020.00312 32432114 PMC7214746

[B54] Snowbarger J. Koganti P. Spruck C. (2024). Evolution of repetitive elements, their roles in homeostasis and human disease, and potential therapeutic applications. Biomolecules 14, 1250. 10.3390/biom14101250 39456183 PMC11506328

[B55] Stapley J. Santure A. W. Dennis S. R. (2015). Transposable elements as agents of rapid adaptation may explain the genetic paradox of invasive species. Mol. Ecol. 24, 2241–2252. 10.1111/mec.13089 25611725

[B56] Stewart N. B. Rogers R. L. (2019). Chromosomal rearrangements as a source of new gene formation in Drosophila yakuba. PLoS Genet. 15, e1008314. 10.1371/journal.pgen.1008314 31545792 PMC6776367

[B69] Tan K. S. Tan S. H. M. (2024). Tussles with mussels: mytiloidean phylogeny revisited (Bivalvia: Pteriomorphia). J. Molluscan Stud. 90. 10.1093/mollus/eyae039

[B57] Thakur J. Packiaraj J. Henikoff S. (2021). Sequence, chromatin and evolution of satellite DNA. Int. J. Mol. Sci. 22, 4309. 10.3390/ijms22094309 33919233 PMC8122249

[B58] Thiriot-Quievreux C. (2002). Review of the literature on bivalve cytogenetics in the last ten years. Cah. Biol. Mar. 43, 17–26.

[B59] Tunjić-Cvitanić M. Pasantes J. J. García-Souto D. Cvitanić T. Plohl M. Šatović-Vukšić E. (2021). Satellitome analysis of the Pacific oyster Crassostrea gigas reveals new pattern of satellite DNA organization, highly scattered across the genome. Int. J. Mol. Sci. 22, 6798. 10.3390/ijms22136798 34202698 PMC8268682

[B60] Tunjić-Cvitanić M. García-Souto D. Pasantes J. J. Šatović-Vukšić E. (2024). Dominance of transposable element-related satDNAs results in great complexity of “satDNA library” and invokes the extension towards “repetitive DNA library.”. Mar. Life Sci. Technol. 6, 236–251. 10.1007/s42995-024-00218-0 38827134 PMC11136912

[B61] Ugarković Đ. Sermek A. Ljubić S. Feliciello I. (2022). Satellite DNAs in health and disease. Genes (Basel) 13, 1154. 10.3390/genes13071154 35885937 PMC9324158

[B62] Uliano-Silva M. Dondero F. Dan Otto T. Costa I. Lima N. C. B. Americo J. A. (2018). A hybrid-hierarchical genome assembly strategy to sequence the invasive golden mussel, Limnoperna fortunei. Gigascience 7, gix128. 10.1093/gigascience/gix128 29267857 PMC5836269

[B63] Vittorazzi S. E. Lourenço L. B. Recco-Pimentel S. M. (2014). Long-time evolution and highly dynamic satellite DNA in leptodactylid and hylodid frogs. BMC Genet. 15, 111. 10.1186/s12863-014-0111-x 25316286 PMC4201667

[B64] Vojvoda Zeljko T. Pavlek M. Meštrović N. Plohl M. (2020). Satellite DNA-like repeats are dispersed throughout the genome of the Pacific oyster Crassostrea gigas carried by helentron non-autonomous mobile elements. Sci. Rep. 10, 15107. 10.1038/s41598-020-71886-y 32934255 PMC7492417

[B65] Wei L. Liu B. Zhang C. Yu Y. Yang X. Dou Q. (2020). Identification and characterization of satellite DNAs in Poa L. Mol. Cytogenet 13, 47. 10.1186/s13039-020-00518-x 33292401 PMC7670724

[B66] Yao Y.-X. Shang X.-P. Yang J. Lin R.-Z. Huai W.-X. Zhao W.-X. (2020). Genetic variation May have promoted the successful colonization of the invasive Gall midge, Obolodiplosis robiniae, in China. Front. Genet. 11, 387. 10.3389/fgene.2020.00387 32362914 PMC7180195

