# Supplementary material for: A diverse satellite DNA repertoire in Limnoperna fortunei: insights into genome structure and chromosomal organization
Source: Front Mol Biosci. 2026 Jan 7;12:1733652. doi: 10.3389/fmolb.2025.1733652 (PMC12818792; doi:10.3389/fmolb.2025.1733652)
Supplement: Supplementary file 3 [file Image1.pdf]

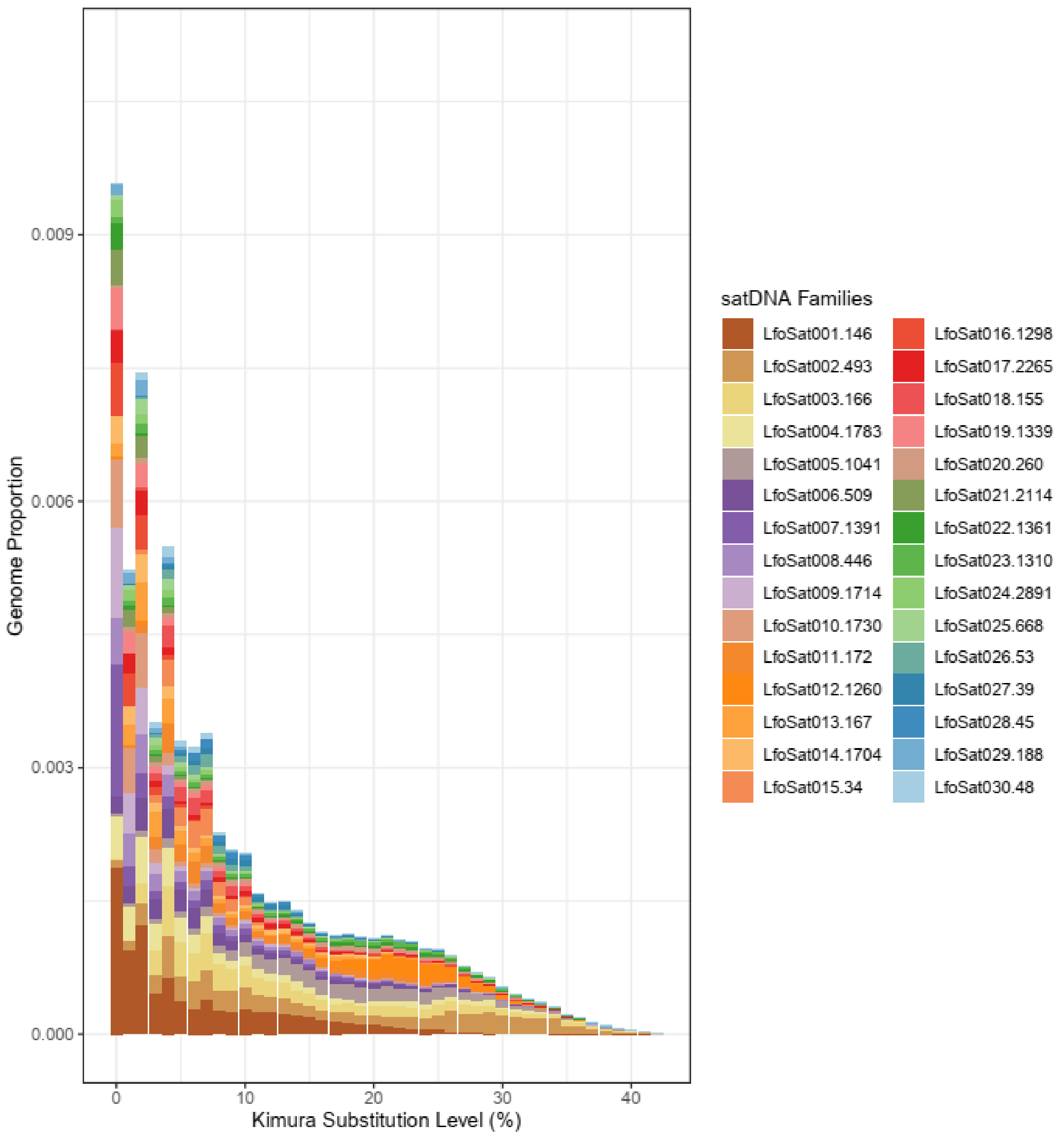

Supplementary Figure S1. Top 30 most satDNAs families in Golden Mussel (*Limnoperna fortunei*) relative to the Kimura substitution level. The graph shows the genomic proportion of the satDNA families at different levels of divergence from the consensus sequences. Nomenclature follows RepeatExplorer2 abundance rankings according to Ruiz-Ruano et al., (2016)
